# In Situ Hydrogel-Forming/Nitric Oxide-Releasing Wound Dressing for Enhanced Antibacterial Activity and Healing in Mice with Infected Wounds

**DOI:** 10.3390/pharmaceutics11100496

**Published:** 2019-09-27

**Authors:** Juho Lee, Shwe Phyu Hlaing, Jiafu Cao, Nurhasni Hasan, Hye-Jin Ahn, Ki-Won Song, Jin-Wook Yoo

**Affiliations:** 1 College of Pharmacy, Pusan National University, Busan 46241, Korea; jhlee2350@gmail.com (J.L.); shwephyuhlaing@gmail.com (S.P.H.); caojiafu1985@163.com (J.C.); hasni1986.nh@gmail.com (N.H.); 2 Department of Organic Material Science and Engineering, Pusan National University, Busan 46241, Korea; hyejin-asu@hanmail.net (H.-J.A.); kwsong@pusan.ac.kr (K.-W.S.)

**Keywords:** in situ hydrogel-forming powder, nitric oxide-releasing formulation, S-nitrosoglutathione (GSNO), antibacterial, wound dressing, wound healing

## Abstract

The eradication of bacteria from wound sites and promotion of healing are essential for treating infected wounds. Nitric oxide (NO) is desirable for these purposes due to its ability to accelerate wound healing and its broad-spectrum antibacterial effects. We developed an in situ hydrogel-forming/NO-releasing powder dressing (NO/GP), which is a powder during storage and forms a hydrogel when applied to wounds, as a novel NO-releasing formulation to treat infected wounds. An NO/GP fine powder (51.5 μm) was fabricated by blending and micronizing S-nitrosoglutathione (GSNO), alginate, pectin, and polyethylene glycol (PEG). NO/GP remained stable for more than four months when stored at 4 or 37 °C. When applied to wounds, NO/GP absorbed wound fluid and immediately converted to a hydrogel. Additionally, wound fluid triggered a NO release from NO/GP for more than 18 h. The rheological properties of hydrogel-transformed NO/GP indicated that NO/GP possesses similar adhesive properties to marketed products (Vaseline). NO/GP resulted in a 6-log reduction in colony forming units (CFUs) of methicillin resistant *Staphylococcus aureus* (MRSA) and *Pseudomonas aeruginosa,* which are representative drug-resistant gram-positive and -negative bacteria, respectively. The promotion of wound healing by NO/GP was demonstrated in mice with full-thickness wounds challenged with MRSA and *P. aeruginosa*. Thus, NO/GP is a promising formulation for the treatment of infected wounds.

## 1. Introduction

Cutaneous wound infections are a global problem whose cost of treatment runs into millions of dollars per year, and these infections can lead to severe complications including sepsis, for which mortality remains around 30% in the United States [1,2]. Wound healing proceeds spontaneously through three sequential phases; inflammation, proliferation, and remodeling [3,4,5,6]. However, when wounds are infected, the healing process is delayed during the inflammation phase, since bacteria induce continuous inflammation at the infected site [7,8]. Thus, eradicating bacteria from the injured site is essential for the treatment of infected wounds.

In recent years, nitric oxide (NO) has gained attention as a novel agent for the treatment of infected wounds because it facilitates wound healing processes such as skin cell proliferation and tissue remodeling, and it also exerts broad-spectrum antibacterial effects [9,10,11,12]. NO enhances re-epithelialization and collagen synthesis during wound healing [5,13]. In addition, NO possesses broad-spectrum antibacterial properties through the formation of reactive nitrogen species such as peroxynitrile, nitrogen dioxide, and dinitrogen trioxide, which can interact with various bacterial proteins, DNA, and enzymes to result in bacterial cell death [14]. Since NO exerts bactericidal effects via multiple biochemical pathways, it exhibits antibacterial effects against drug-resistant bacteria, including methicillin resistant *Staphylococcus aureus* (MRSA) [15]. Moreover, NO does not develop drug resistance due to its diverse antibacterial mechanisms, which require multiple mutations for resistance to develop [16]. However, despite these beneficial effects of NO, its clinical application remains challenging because of its short half-life and gaseous nature. Therefore, the development of an NO-releasing formulation with a sustained NO-release and good storage stability is required.

We hypothesized that a powder dressing that forms a hydrogel in situ maintains a powder state during storage and converts to hydrogel immediately when applied to wounds could be an ideal NO-releasing formulation for the treatment of infected wounds. An in situ hydrogel-forming/NO-releasing powder dressing possesses the benefits of both powders and hydrogels. Since a water-free powder ensures high storage stability, the stability of easily hydrolyzable pharmaceutical ingredients can be markedly improved. When applied to the wound site, an in situ hydrogel-forming powder dressing can fit the irregular surface of the wound without wrinkles or fluting [17,18,19]. In addition, in situ hydrogel-forming powders are easy to apply to bendable areas of the human body, such as elbows, finger joints, ankles, or wide wound beds resulting from burn and pressure ulcers. Once applied to the wound, they can absorb the wound fluid and form an adhesive hydrogel, which can protect the wound site from the external environment and maintain a humid environment to aid wound healing [20,21]. At the same time, a NO release triggered by the absorbed wound fluid may assist wound healing via the elimination of bacteria and stimulation of wound-healing processes [22].

In this study, a novel in situ hydrogel-forming/NO-releasing powder dressing (NO/GP) possessing the benefits of both powders and hydrogels was developed using S-nitrosoglutathione (GSNO), alginate, pectin, and polyethylene glycol (PEG) for the treatment of infected wounds. GSNO is a widely used endogenous NO donor that can generate NO over several hours under physiological conditions, which may be beneficial properties for NO releasing wound dressings [23]. Sodium alginate was selected to form a bioadhesive hydrogel. In addition, it can absorb wound exudate up to approximately 20-times its weight [24]. Pectin was used to form a hydrogel structure and accelerate the powder-to-hydrogel transition [19]. PEG was added to modulate the ability of the formulation to uptake fluid to prevent potential wound drying caused by the excessive absorption of exudate. Following the characterization of NO/GP, the storage stability, in situ hydrogel-forming ability, rheological properties, and NO-release profiles were evaluated in a series of in vitro and in vivo experiments. The antibacterial effects of NO/GP were examined against MRSA and *Pseudomonas aeruginosa*, which are drug-resistant gram-positive and -negative bacteria, respectively. The in vivo therapeutic effects of NO/GP were evaluated using a bacteria-challenged full-thickness wound mouse model.

## 2. Materials and Methods 

### 2.1. Materials

Glutathione (reduced form) was purchased from Wako Pure Chemical (Osaka, Japan). Pectin and agar were purchased from Yakuri Co., Ltd. (Osaka, Japan). Sodium alginate, PEG (average molecular weight: 8000), sodium nitrite, crystal violet, Lugol’s solution, 2,2,2 tribromoethanol, *tert*-amyl alcohol, Mayer’s hematoxylin, and eosin-Y disodium were purchased from Sigma-Aldrich (St. Louis, MO, USA). Bacto™ tryptic soy broth (TSB) and Difco™ cetrimide-agar media were purchased from BD Biosciences (San Jose, CA, USA). A Twort’s Gram stain set was purchased from Newcomer Supply (Middleton, WI, USA). Masson’s trichrome stain kit was purchased from Abcam (Cambridge, MA, USA). The LIVE/DEAD^®^ *Bac*Light™ bacterial viability kit was purchased from Thermo Fisher Scientific (Waltham, MA, USA). All other reagents and solvents were of the highest analytical grade commercially available.

### 2.2. GSNO Synthesis

GSNO was synthesized following a previously reported method with some modifications [25]. Briefly, sodium nitrite and reduced glutathione were added to a cold HCl solution with stirring for 40 min in an ice bath (the final concentration of NaNO_2_, glutathione, and HCl was 0.625 M). To precipitate GSNO, acetone was added and stirred for 20 min. The precipitate was collected by filtration and washed once with 80% acetone, twice with 100% acetone, and three times with diethyl ether. After drying, GSNO was stored in a −20 °C refrigerator for subsequent experiments.

### 2.3. Preparation and Characterization of the NO-Releasing In Situ Hydrogel-Forming Powder

To prepare the in situ hydrogel-forming agent, sodium alginate, pectin, and PEG were micronized, sieved (90 μm), and blended. The ratio of alginate, pectin, and PEG used for the in situ hydrogel-forming powder (GP) was optimized with sodium alginate:pectin:PEG at a 2:1:6 ratio in several pilot studies. To prepare the NO-releasing in situ hydrogel-forming powder (NO/GP), GSNO was added to the GP (final content of GSNO was 4 wt %). GSNO was sieved before blending with GP to eliminate the GSNO aggregates, which could induce undesirable effects in the content uniformity. NO/GP was stored in a −20 °C refrigerator.

NO/GP was characterized by determining particle size, GSNO content, content homogeneity, and powder flowability. Briefly, the particle sizes of NO/GP and the non-micronized mixture were analyzed by ImageJ software (National Institutes of Health, Bethesda, MA, USA) using microscopic images. More than 100 particles were used to assess particle size. GSNO contents were determined using 10 samples of each NO/GP and non-micronized mixture, which were dissolved in distilled water (DW), and then the absorbance was detected using a UV/Vis spectrophotometer (U-5100, Hitachi, Tokyo, Japan) at 335 nm. From the absorbance values, GSNO content and relative standard deviation (RSD) were calculated to investigate GSNO content and content homogeneity, respectively. Powder flowability was evaluated as previously reported, with some modifications [26]. Briefly, 40 mg of NP/GP was loaded in a 1 mL syringe and tapped until no change in volume was detected. The bulk and tapped density were calculated from the tapped and untapped volume of NO/GP, and the Hausner ratio was calculated based on the tap density/bulk density ratio.

### 2.4. Rheological Properties of NO/GP in Hydrogel Form

The rheological properties of the hydrogel-form of NO/GP were evaluated as previously reported, with some modification [27,28,29]. The steady shear and dynamic viscoelastic properties of the hydrogel-form of NO/GP were measured using a strain-controlled rheometer (Advanced Rheometric Expansion System [ARES], Rheometric Scientific, Piscataway, NJ, USA) equipped with a parallel-plate fixture with a radius of 12.5 mm and a gap size of 1.0 mm. All rheological measurements were performed at a fixed temperature of 37 °C over a wide range of shear rates and strain amplitudes. In this study, simulated wound fluid (SWF) [30,31] was used to induce NO/GP swelling. The SWF consisted of 0.64% NaCl, 0.22% KCl, 2.5% NaHCO_3_, and 0.35% NaH_2_PO_4_ in double distilled water with pH 7.4. Three different conditions of NO/GP in hydrogel form (NO/GP that absorbed 200%, 350%, and 500% of SWF absorbed per weight) were examined with SWF. Before initiating the experiments, 2, 3.5, or 5 mL of SWF were added to 1 g of NO/GP and mixed well to obtain the homogeneous hydrogel. In all experiments, a fresh sample was used and rested for 15 min after loading to allow for material relaxation and temperature equilibration. To evaluate the steady shear flow behaviors of the hydrogel-form of NO/GP, steady rate-sweep tests were performed over a range of shear rates from 1 to 1000 s^−1^ with a logarithmically increasing scale. Next, strain-sweep tests were conducted to investigate both the linear viscoelastic region and nonlinear viscoelastic behavior over a strain amplitude range of 0.0625%–500% at a fixed angular frequency of 10 rad/s.

### 2.5. Storage Stability

The storage stability of NO/GP was evaluated by determining GSNO degradation under two temperature conditions: 4 and 37 °C. Two milligrams of NO/GP were placed in each tube, which were then stored in either a 4 °C refrigerator or a 37 °C incubator. At previously set time points, three tubes from each group were sampled, and the absorbance at 335 nm was measured to determine the GSNO content following dilution with DW.

### 2.6. Behavior of NO/GP After Exposure to Wound Fluid

#### 2.6.1. Morphological Changes of NO/GP at the Wound Site

All animal experiments in this study have been reviewed and approved by the Pusan National University Institutional Animal Care and Use Committee (PNU-IACUC) on 01 February 2018 (Approval Number: PNU-2018-1800). To investigate the ability of NO/GP to form hydrogel in situ, macroscopic images of wounds treated with NO/GP were taken at each time point. Briefly, imprinting control region (ICR) mice (7 weeks old, male, Samtako Bio Korea) were purchased and acclimated for 7 days. To induce anesthesia, 0.5–0.6 mg/g of avertin (tribromoethanol) were administered intraperitoneally. Then, hair was removed from the dorsal side of the mouse by electric trimmers and hair removal cream (Veet for sensitive skin, Reckitt Benckiser, France). After hair removal, a full-thickness wound was created on the dorsal area of the mouse via an 8 mm diameter disposable biopsy punch (Kai medical, Japan). Macroscopic images were taken at each time point following treatment of the wound with 28.5 mg NO/GP.

#### 2.6.2. Fluid Uptake Ability

The ability of NO/GP to uptake fluid was measured as previously described, with some modifications [26,32]. A three-station Franz diffusion cell apparatus (PermeGear, Inc., Hellertown, PA, USA) was used to measure the water uptake ability. A regenerated cellulose membrane (pore size = 0.45 μm) was placed between the donor and receiver compartments. In the receiver compartment, SWF was filled and thermostated at 37 °C. After SWF was loaded into the receiver compartment, 40 mg of NO/GP were placed on the regenerated cellulose membrane (donor compartment). The amount of SWF was maintained at 8 mL. At each time point, the weight of the donor compartment was measured to calculate the amount of absorbed fluid.

#### 2.6.3. NO Release from NO/GP

The NO release from NO/GP was calculated by measuring the GSNO decomposition. The amount of GSNO remaining was determined using a UV/Vis spectrophotometer at a wavelength of 335 nm. Fifty milligrams of NO/GP powder were placed in a 2 mL microtube, and different amounts of SWF were added to mimic swelling (NO/GP that absorbed 200%, 350%, and 500% of SWF per weight). All microtubes were placed in a 37 °C incubator. At the set time points, the remaining GSNO was measured by determining the absorbance of the supernatant at 335 nm after dilution and centrifugation. The NO released from NO/GP at each time point ([NO]_t_) was calculated using an Equation (1) ([GSNO]_0_: Initial GSNO concentration; [GSNO]_t_: GSNO concentration at each time point) [25,33].

[NO]_t_ = [GSNO]_0_ − [GSNO]_t_ (1)

### 2.7. Antibacterial Assay

The bactericidal effect of NO/GP was evaluated against *P. aeruginosa* PAO1 (wild-type prototroph) [34] and MRSA (USA 300) [35]. Each pathogen was incubated overnight in TSB at 37 °C, and the bacterial suspension was adjusted with TSB media to approximately 10^8^ colony forming unit (CFU)/mL until the optical density at 600 nm reached 0.15–0.2 (0.5 of the McFarland scale) [36]. The adjusted bacterial suspension (100 μL) was inoculated into each tube. Then, 28.5 mg of NO/GP and GP were added to each well and incubated for 24 h at 37 °C. To calculate the number of living bacterial cells, tubes were diluted with an additional 1.9 mL of the TSB medium. After serial dilution, 100 μL of each aliquot were plated on the TSB agar and incubated for 24 h at 37 °C. CFUs were determined by counting the colonies on the agar plates after incubation. To visualize the antibacterial activity of NO/GP, bacteria treated with or without GP and NO/GP were stained with SYTO9 (Thermo Fisher Scientific, Waltham, MA, USA) (final concentration was 66.8 μM) for 15 min. After incubation, bacteria were collected by centrifugation at 3000 *g* for 10 min. Each sample was washed three times and resuspended in 5 mL of normal saline. Green fluorescence from stained bacteria was imaged by an in vivo imaging system (FOBI, Neoscience, Suwon, Korea). For confocal laser scanning microscopy, bacteria were washed three times with normal saline after 24 h incubation with or without GP and NO/GP. Then, the bacteria were stained with SYTO 9 dye and propidium iodide (LIVE/DEAD^®^ *Bac*Light™ bacterial viability kit) according to the manufacturer’s protocol. Images were obtained at 20× magnification using the LSM 800 (Carl Zeiss, Oberkochen, Germany).

### 2.8. In Vivo Wound Healing Study in a Bacteria-Challenged Full Thickness Wound Model

#### 2.8.1. Evaluation of Wound Size Reduction

In this study, mouse models of *P. aeruginosa-* and MRSA-challenged full thickness wounds were used to evaluate the ability of NO/GP to heal infected wounds. For *P. aeruginosa*-challenged wound healing study, 8 mm-sized wounds were created using above-mentioned method. Then, 10^9^ CFU of *P. aeruginosa* suspension was inoculated at the wound site. Each wound was covered with Tegaderm film and fastened by surgical tape (Durapore™, 3M) for protection. Mice were then incubated for 2 days with no treatment for wound infection. Two days after wounding, mice were treated with 28.5 mg of NO/GP and GSNO-free NO/GP every 2 days. Untreated mice were used as a control group (changing Tegaderm film and surgical tape only). Photographs were obtained every 2 days, and the size of the wound was analyzed by ImageJ software. For the MRSA-challenged wound healing study, a 2 × 10^6^ CFU of MRSA suspension was used instead of *P. aeruginosa,* and the procedure described above was followed.

#### 2.8.2. Quantification of *P. aeruginosa* at the Wound Site

In the *P. aeruginosa*-challenged wound study, bacteria were quantified at the wound as previously reported, with some modifications [37]. Briefly, wound samples were harvested from representative mice on predetermined days with an 8 mm diameter biopsy punch. Each sample was placed in 1 mL of PBS, chopped, and sonicated to detach bacteria from the tissue samples. Then, 100 μL of each tissue-bacteria suspension was plated on an agar plate after serial dilution (1:10). To quantify the amount of *P. aeruginosa* at the wound site, a cetrimide-agar plate was used as a pseudomonas-selective media [38]. CFUs were determined by counting the colonies on the agar plates after 24 h of incubation.

#### 2.8.3. Histological Examination

In the *P. aeruginosa*-challenged wound healing study, mice were euthanized 14 days after the initiation of drug treatment, and each wound site was sampled with an 8 mm diameter biopsy punch. Each sample was immediately immersed in 10% buffered formalin for fixation. Fixed wound samples were placed in paraffin blocks, sectioned to obtain 5 μm wound samples, and prepared for hematoxylin and eosin (H&E) staining, Twort’s Gram staining, and Masson’s trichrome staining. Each staining procedure was performed according to the manufacturer’s protocol with some modifications. After staining, each slide was photographed using a light microscope at 20× magnification for H&E and Masson’s trichrome staining, as well as 100× for Twort’s gram staining.

### 2.9. Statistical Analysis

The statistical analysis was performed using a one-way analysis of variance (ANOVA) with a Bonferroni posttest in GraphPad Prism 5.0 (GraphPad Software, Inc., La Jolla, CA, USA). *p*-values less than 0.05 were considered statistically significant.

## 3. Results and Discussion

### 3.1. Powder Characterization

**Figure 1:**
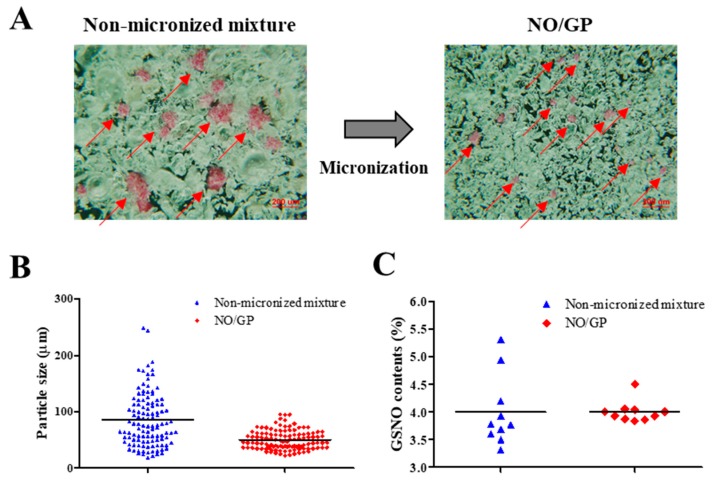
Characterization of NO/GP in powder form. Microscopic images (**A**), particle size distribution (*n* > 100) (**B**), and S-nitrosoglutathione (GSNO) contents of non-micronized mixture and NO/GP (*n* = 10) (**C**). Red arrows indicate GSNO particles (pink particles).

NO/GP was successfully prepared by micronizing, sieving, and blending GSNO, sodium alginate, pectin, and PEG. To examine the homogeneous fabrication of NO/GP, particle size distribution was analyzed using more than 100 particles, and GSNO contents were measured from 10 different samples. As shown in Figure 1A, pink GSNO particles were homogeneously dispersed in NO/GP. After micronizing, the average particle size and standard deviation decreased (from 85.2 ± 47.3 to 51.5 ± 22 μm, respectively) (Table 1, Figure 1A,B). In addition, the RSD of GSNO contents was reduced (16.06–4.77) due to the removal of large GSNO particles which interfere with content homogeneity (Table 1, Figure 1C). Flowability was measured to investigate the movement of NO/GP at the wound site before conversion to hydrogel. The flowability of in situ hydrogel-forming powders should be low because highly flowable powders can be easily cleared from the wound site prior to hydrogel formation. In this experiment, the flowability of NO/GP was expressed by the Hausner ratio. After the blending and micronizing process, the Hausner ratio of NO/GP increased from 1.34 to 1.97 (Table 1). Due to the decreased particle size, flowability decreased after micronization. In general, powders with a Hausner ratio exceeding 1.6 possess extremely poor flowability [39]. Low flowability can prevent the removal of the powder and is essential for the in situ hydrogel-forming powder system; the powder should remain on the wound site until it is converted to a hydrogel. For this reason, the Hausner ratios of previously developed powder dressings are around 1.7 [18,19,26], as this ratio allows the powders to resist the flow. Therefore, NO/GPs with a Hausner ratio of 1.97 could also resist flow away from the wound site before hydrogel formation.

### 3.2. Rheological Properties of NO/GP in Hydrogel Form

**Figure 2:**
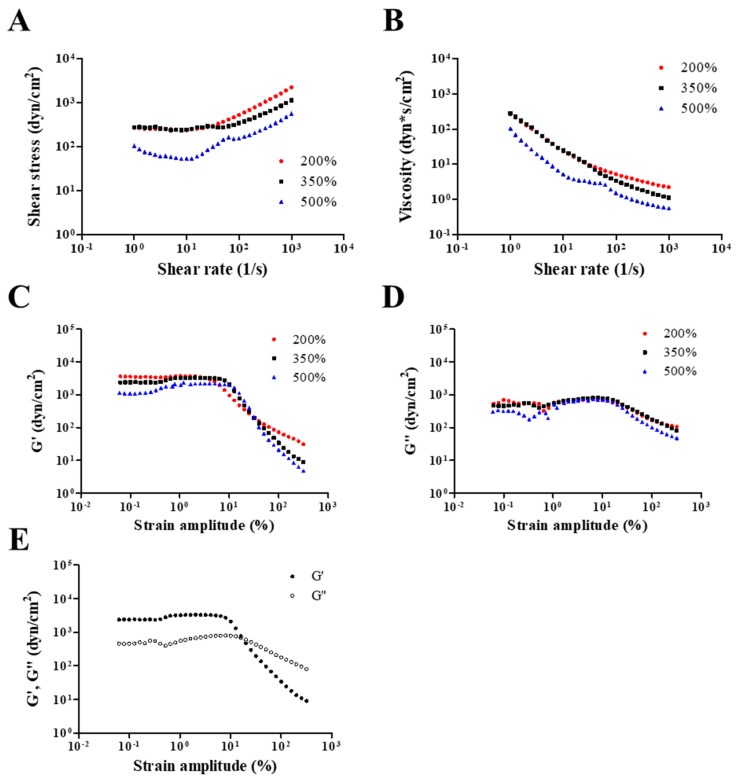
Rheological properties of NO/GP in hydrogel form. Shear stress and viscosity (**A** and **B**, respectively) of NO/GP following absorption of 200%, 350%, and 500% of simulated wound fluid (SWF) per weight as a function of shear rate. Storage modulus (*G’*) and loss modulus (*G”*) of each swollen condition as a function of strain amplitude (**C** and **D**, respectively). *G’* and *G”* of 350% swollen NO/GP hydrogel as a function of strain amplitude (**E**). Strain-sweep tests were performed at a fixed angular frequency of 10 rad/s.

Rheological properties, including viscosity, storage modulus, and loss modulus, were evaluated to investigate the adhesiveness of NO/GP in hydrogel form against various steady shear strains or oscillatory shear strains. Adhesiveness is an important factor of hydrogel dressings because hydrogels should adhere to the damaged site to protect the wound and maintain humid conditions, which is an essential role of hydrogel dressings. Figure 2A,B show the dependence of shear stress and steady shear viscosity on shear rate for NO/GP following the absorption of 200%, 350%, and 500% SWF per weight. In all steady rate-sweep tests, the shear stress tended to level off and approach a limiting constant value (usually referred to as “yield stress”) as the shear rate approached zero. Yield stress plays an important role in predicting the adhesiveness of semi-solid formulations because stress is related to the level of internal structures that can exhibit resistance to flow. Steady shear viscosity decreased sharply as the shear rate increased, indicating that NO/GP exhibited a marked non-Newtonian shear-thinning flow behavior. In a previous study, petroleum jelly, which is a widely used and marketed product, demonstrated similar behavior in the 350% swollen condition, and the values of shear stress and steady shear viscosity were also similar to those of the swollen condition [40]. These results indicate that the NO/GP hydrogel could maintain a gel-like structure and resist small shear stress such as gravitational force or brushing against clothes. Figure 2C,D,E indicate the storage modulus, *G’*, and loss modulus, *G”*, as a function of strain amplitude under three different swelling conditions of NO/GP hydrogels with a fixed angular frequency of 10 rad/s. The storage modulus was found to be larger than the loss modulus within a relatively smaller strain amplitude, indicating that the rheological behavior in this region is dominated by an elastic (solid-like) rather than a viscous (liquid-like) property. However, as the strain amplitude gradually increased, viscous behavior became superior to elastic behavior because the storage modulus demonstrated a sharper decrease with increasing strain amplitude compared with the loss modulus. These results indicate that adhesiveness to a relatively large imposed deformation (such as scrubbing motion) is weakened; therefore, the NO/GP hydrogels could easily flow and be removed from the wound site.

### 3.3. Storage Stability

**Figure 3:**
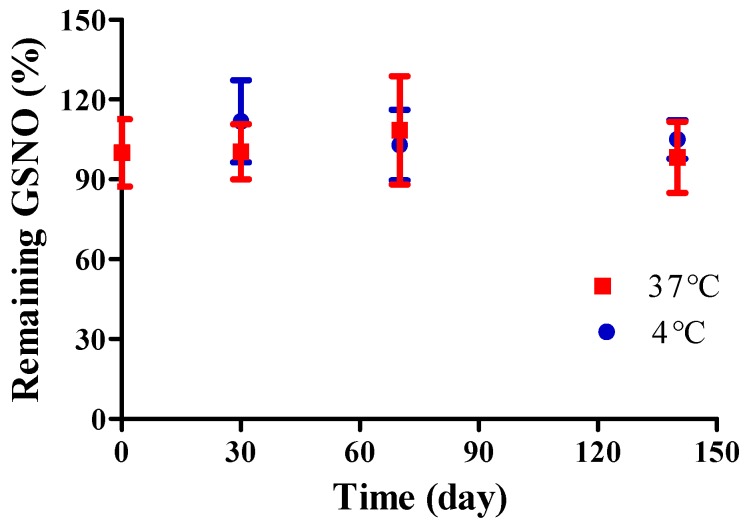
Storage stability of NO/GP at 4 (blue) and 37 °C (red). Remaining GSNO percentage compared with that of initial NO/GP was calculated to evaluate storage stability. Each point represents the mean ± standard deviation (*n* = 3).

The storage stability of NO/GP was evaluated by detecting GSNO decomposition under two temperature conditions (4 and 37 °C). Since GSNO can be easily degraded by hydrolysis, storage stability is an important factor in the development of GSNO-containing formulations. Though several GSNO-containing hydrogel dressings have been developed to accelerate wound healing [41,42,43,44], those formulations did not present long-term stability because GSNO hydrolysis is inevitable in water-containing formulations. Conversely, no significant GSNO decomposition was noted in NO/GP up to 140 days under both the 4 and 37 °C conditions (Figure 3). Since GSNO in the NO/GP remained in a powder state, which was a water-free condition during storage, the hydrolysis of GSNO was prevented. Thus, NO/GP could remain stable whilst in storage without any concerns relating to GSNO degradation. In addition, there were no significant changes in particle size, Hausner ration and rheological properties during the storage period (data not shown).

### 3.4. Behavior of NO/GP After Exposure to Wound Fluid

#### 3.4.1. Morphological Changes in NO/GP at the Wound Site

**Figure 4:**
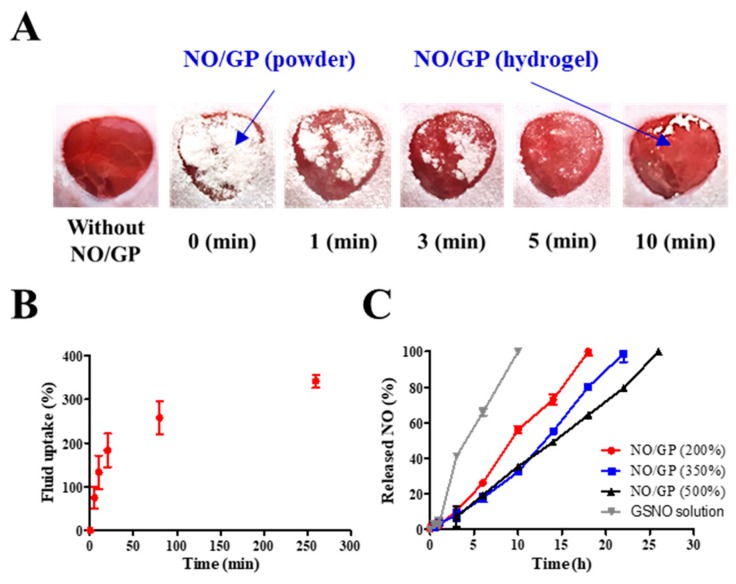
Behavior of NO/GP following exposure to wound fluid. Morphological changes in NO/GP applied to the mouse full-thickness wound model (**A**). Fluid uptake profile of NO/GP (amount of absorbed SWF is presented as the percent per initial NO/GP powder weight) (**B**). NO release profiles of NO/GP under different swelling conditions and the GSNO solution at the equivalent concentration to 350% swollen NO/GP (**C**). Each point represents the mean ± standard deviation (*n* = 3).

To investigate the ability of NO/GP to form hydrogel in situ, morphological changes in NO/GP were observed following application of 28.5 mg of NO/GP to the full-thickness wounds in mice. The amount of NO/GP was sufficient to cover 1 cm^3^ of a full-thickness wound. As shown in Figure 4A, following its application to the wound, the NO/GP powder was immediately converted to a glittering hydrogel, and more than 50% of the NO/GP powders converted to a hydrogel within 1 min. All of the NO/GP applied was converted to hydrogel within 10 min. After 10 min, no morphological changes in NO/GP were observed due to the completion of the hydrogel structure.

#### 3.4.2. Fluid Uptake Ability

Since hydrogel formation is initiated by the absorption of wound fluid, an investigation of fluid uptake ability is essential for evaluation of powder dressings that form hydrogels in situ. To evaluate the fluid uptake ability of NO/GP, the dressings were exposed to SWF at 37 °C, and the amount of absorbed fluid was calculated by measuring the change in weight of NO/GP. The amount of absorbed fluid was presented as a percentage of weight gained by fluid uptake per initial NO/GP. As shown in Figure 4B, NO/GP absorbed SWF rapidly, and around 200% of SWF was absorbed within 20 min. After the initial rapid absorption of the SWF, the rate of fluid uptake by NO/GP was decreased, and NO/GP absorbed up to 375% of SWF in 270 min. After that, no further significant fluid absorption was observed during the experiment. In the initial state, the swellable polymers in NO/GP (pectin and alginate) absorbed SWF and rapidly formed a hydrogel structure. Following hydrogel formation, SWF was slowly captured in the intermolecular space of the hydrogel structure, because hydrophilic polymers in NO/GP are able to trap SWF by hydrogen bonding between polymers and water molecules. Finally, since the intermolecular space was filled with SWF, no more fluid could be absorbed. Since NO/GP could efficiently absorb fluid, hydrogel formation and subsequent NO release was initiated rapidly.

#### 3.4.3. NO Release from NO/GP

Since NO released from GSNO in NO/GP exerts therapeutic effects, the NO release profiles of NO/GP were investigated by exposing NO/GP to SWF at 37 °C. The NO release from NO/GP was investigated under three swollen conditions (200%, 350%, and 500% of SWF per initial NO/GP weight) that represented an amount of low, medium and high wound exudate, respectively. As GSNO generates NO via hydrolytic cleavage of the S–N bond, the NO release from NO/GP is initiated by wound fluid. Thus, the amount of NO released from NO/GP was calculated by measuring GSNO degradation (released NO = initial GSNO − remaining GSNO). Regardless of swelling, NO/GP exhibited a linear NO release without a burst release (Figure 4C). In addition, lower levels of NO/GP swelling resulted in a slightly faster NO release rate compared with higher levels of swelling, and NO was released up to 18, 22, and 26 h in the 200%, 350%, and 500% conditions, respectively. Due to the presence of polymeric compounds (alginate, pectin, and PEG) in the NO/GP hydrogel structure, GSNO molecules or NO radicals were surrounded by polymeric molecules which restrict the diffusion of radicals in what is termed the “cage effect” [45,46]. Compared with the GSNO solution, NO/GP presented a prolonged NO-release profile, which was due to the restricted diffusion of radicals over the cage (NO was released 100% within 12 h at the GSNO concentration equivalent to 350% swollen NO/GP). The release profiles different under different levels of swelling because the rate of NO release from GSNO was affected by its initial concentration; thus, the higher initial concentration resulted in faster NO release due to the increased amount of radicals contributing to the degradation of GSNO molecules [47]. Therefore, 200% SWF added to the NO/GP group (high GSNO concentration) resulted in a faster NO release compared with the 350% and 500% groups. In addition, since NO/GP exhibited a linear NO release under all levels of swelling, this indicates that dressings can be changed any time without concerns of toxicity caused by burst-released NO.

### 3.5. In Vitro Antibacterial Assay

**Figure 5:**
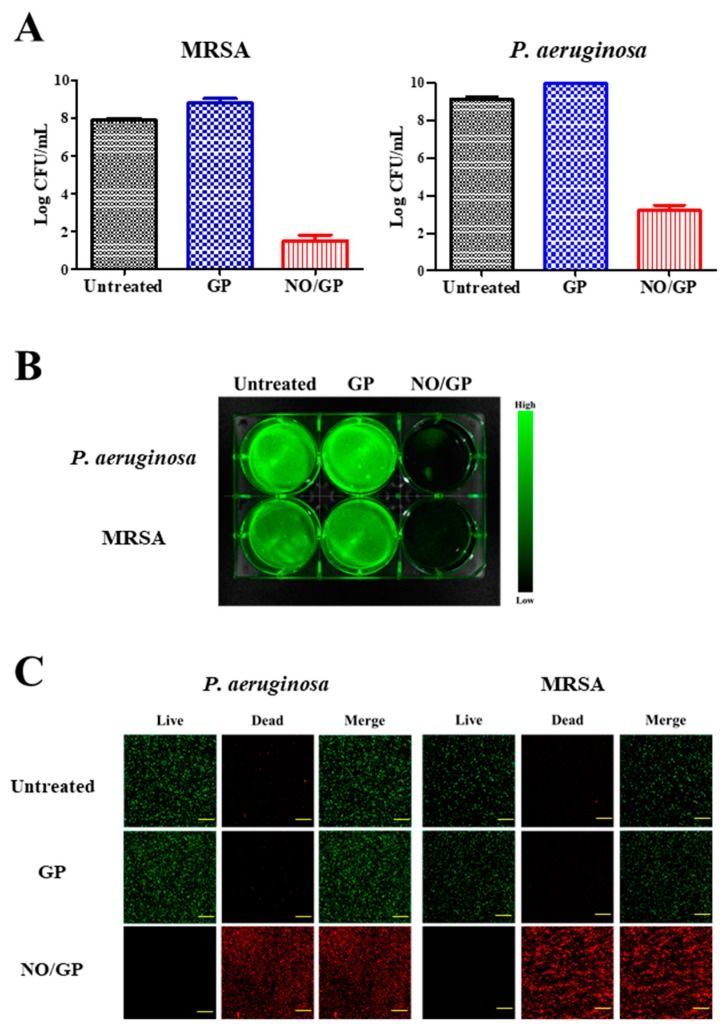
Antibacterial effects of NO/GP. Antibacterial effects of NO/GP against *Pseudomonas aeruginosa* and methicillin-resistant *Staphylococcus aureus* (MRSA) by the colony forming unit (CFU) method. The results are presented as the mean ± standard deviation (*n* = 3) (**A**). Visualization of bacteria by SYTO9 staining. Green fluorescence indicates the presence of live bacteria (**B**). Confocal laser scanning microscopic images of cells treated and untreated with GP and NO/GP. Green fluorescence indicates live bacteria and red fluorescence indicates dead bacteria. Images were taken at 20× magnification. Scale bar represents 50 μm (**C**).

Antibacterial efficacy is the most important characteristic of a dressing for the treatment of infected wounds. To investigate the antibacterial activity of NO/GP, an in vitro antibacterial assay was performed using the CFU method against MRSA and *P. aeruginosa*, which are representative drug-resistant gram-positive and -negative bacteria. Following incubation for 24 h with or without NO/GP in TSB media, a 6-log reduction in bacterial CFUs was observed in the NO/GP-treated group compared to the GP-treated group against both MRSA and *P. aeruginosa* (Figure 5A). After CFU examination, the antibacterial activity of NO/GP was visualized by staining with SYTO 9, which is a green fluorescence dye that can stain bacterial DNA. Since only living bacteria were collected by centrifugation, green fluorescence indicated the presence of live bacteria. As shown in Figure 5B, distinct green fluorescence was detected in the untreated and GP-treated groups for both MRSA and *P. aeruginosa*. Furthermore, signals from the NO/GP-treated group were significantly reduced, owing to the high number of bacteria killed by NO in both the MRSA and *P. aeruginosa* groups. The antibacterial effect of NO/GP was also examined via confocal microscopy using the LIVE/DEAD^®^ *Bac*Light™ bacterial viability kit. Since propidium iodide can only penetrate damaged bacterial membranes, living bacteria were stained with SYTO 9 (green fluorescence) and damaged bacteria were stained with propidium iodide (red fluorescence). As shown in Figure 5C, confocal images of the GP-treated and untreated groups exhibited distinct green fluorescence, while those of the NO/GP-treated group exhibited strong red fluorescence. This indicates that most of the bacteria survived in the GP-treated and untreated groups, while few bacteria survived in the NO/GP-treated group. Since NO possesses broad-spectrum antibacterial effects and NO/GP can release NO in a sustained manner, these results indicate that NO/GP exhibited significant bactericidal activity against both gram-negative *P. aeruginosa* and gram-positive MRSA without bacterial re-growth for 24 h. Broad-spectrum antibacterial and potent bactericidal effects against drug-resistant bacteria are essential for the treatment of infected wounds, since the infection of cutaneous wounds by drug-resistant bacteria is increasing and it is hard to immediately distinguish bacterial species. Moreover, the multiple antibacterial mechanisms of NO may prevent the emergence of NO-resistant bacteria [16]. Thus, these findings indicate that NO/GP possesses desirable antibacterial properties and may be beneficial for the treatment of infected wounds.

### 3.6. In Vivo Wound Healing Study

#### 3.6.1. Evaluation of Wound Size Reduction Effect

**Figure 6:**
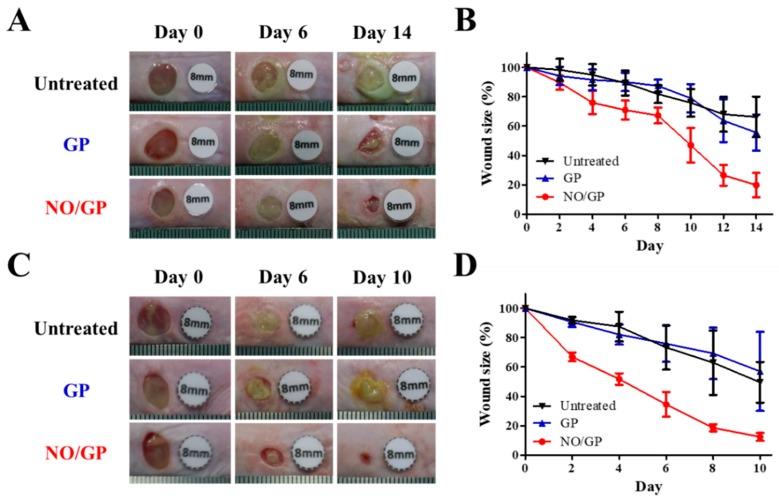
Representative macroscopic images of *P. aeruginosa*-challenged full-thickness wounds in mice 0, 6, and 14 days after treatment initiation (**A**). Changes in wound size in mice with *P. aeruginosa*-challenged full-thickness wounds (**B**). Representative macroscopic images of MRSA-challenged full-thickness wounds in mice 0, 6, and 14 days after treatment initiation (**C**). Changes in wound size in mice with MRSA-challenged full-thickness wounds (**D**). Values are presented as the mean ± standard deviation (*n* = 4).

The therapeutic effects of NO/GP were evaluated in mice using the bacteria-challenged full-thickness wound model. The acceleration of infected wound recovery with NO/GP was evaluated by observing morphological changes in the wound and measuring wound size change every 2 days. In both *P. aeruginosa*- and MRSA-challenged full-thickness wound models, the NO/GP treatment resulted in a significant reduction in wound size compared with GP treatment and no treatment after 4 days (Figure 6). In NO/GP-treated groups, wound size was reduced to less than 20% of the initial size 14 and 8 days after treatment initiation in the *P. aeruginosa*- and MRSA-challenged models, respectively. Conversely, in the GP treated groups, no significant acceleration of wound healing was observed in either the *P. aeruginosa*- or MRSA-challenged models compared to the untreated groups. Accelerated wound healing in the NO/GP groups can be attributed to the action of NO released from GSNO in NO/GP [22]. In particular, broad and potent antibacterial effects could effectively eradicate infection with gram-positive or -negative bacteria [48,49]. Moreover, NO facilitates wound healing by promoting fibroblast proliferation, collagen formation, and tissue remodeling [13,50]. Therefore, NO/GP may facilitate wound healing in *P. aeruginosa*- and MRSA-challenged full-thickness wounds in mice.

#### 3.6.2. Quantification of P. aeruginosa at the Wound Site

**Figure 7:**
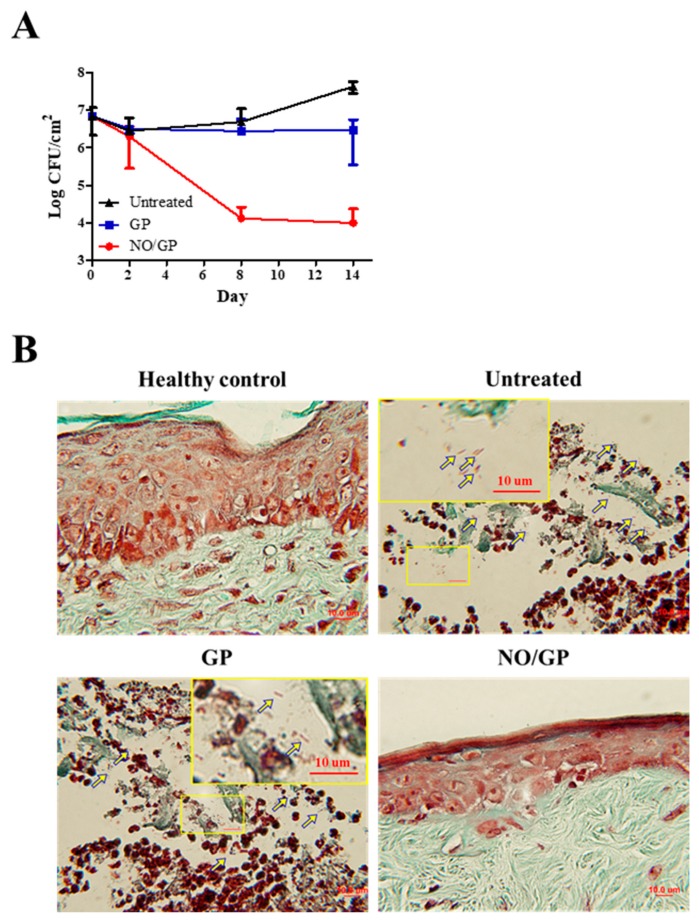
Bacterial quantification at the wound site (*n* = 3) (**A**). Twort’s gram staining of wound samples from mice treated with or without GP or NO/GP. The epidermal region of the tissue samples was imaged with a microscope at a magnification of 100×. Arrows indicate *P. aeruginosa* (1–2 μm sized, rod-shaped, and brown). Scale bar represents 10 μm (**B**).

To investigate the in vivo antibacterial effects of NO/GP, wound samples were harvested 2, 8, and 14 days after treatment initiation, and CFUs were assessed with *Pseudomonas*-selective agar plates, which exclude other bacterial species. As shown in Figure 7A, there was no decrease in the number of *P. aeruginosa* 2 days after treatment initiation; however, on day 8, significant bactericidal effects were observed in the NO/GP-treated group (around 3-log CFU reduction). Only 4.1 and 4.8 CFU/cm^2^ *P. aeruginosa* were observed in the NO/GP-treated group, while 6.7 and 7.6 CFU/cm^2^ and 6.4 and 6.5 CFU/cm^2^ *P. aeruginosa* were observed in untreated and GP-treated groups 8 and 14 days after treatment initiation, respectively. To visualize the antibacterial effects in vivo, Twort’s Gram-staining was performed. Because *P. aeruginosa* is a rod-shaped gram-negative bacteria 1–2 μm in size, it can be detected by Twort’s gram staining in tissue samples containing more than 10^5^ CFU (low levels of bacteria are hard to detect by Gram staining) [51,52]. Fourteen days after treatment initiation, rod-shaped, brown-colored bacteria (*P. aeruginosa*) were observed in GP-treated and -untreated groups in the damaged epidermal region, indicating that at least 10^5^ CFU *P. aeruginosa* was present in the samples (Figure 7B). Conversely, no bacteria were observed in the NO/GP-treated group or in the healthy control. Since NO released from NO/GP was able to efficiently eradicate *P. aeruginosa* from infected wound sites, wound healing may have occurred subsequent to inflammation. Furthermore, high numbers of *P. aeruginosa* in GP-treated and untreated groups resulted in consistent inflammation and, consequently, in impaired re-epithelization.

#### 3.6.3. Histological Examination

**Figure 8:**
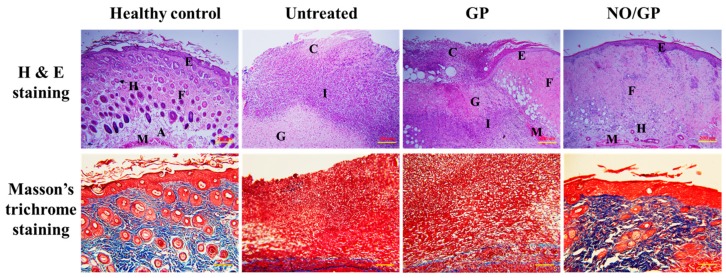
H&E (hematoxylin and eosin) and Masson’s trichrome straining of wound tissues from *P. aeruginosa*-challenged full-thickness wound mice threated with or without GP or NO/GP for 14 days. A: Adipose tissue; C: Cell debris; E: Epidermis; F: Fibrous tissue; G: Granulation tissue; H: Hair follicle; I: Immune cells; M: Muscle. Scale bar represents 200 and 100 μm for the H&E and Masson’s trichrome images, respectively.

Tissue regeneration and collagen synthesis in full thickness wounds challenged with *P. aeruginosa* were evaluated by H&E and Masson’s trichrome staining. Fourteen days after the initiation of drug treatment, more organized skin morphology and higher collagen abundance were observed in the NO/GP-treated group compared with the GP-treated and untreated groups (Figure 8). Well-differentiated epidermis was observed in the NO/GP-treated group, whilst damaged epidermis was observed in the GP-treated and untreated groups following H&E staining. In addition, skin cells, such as keratinocytes and fibroblasts, were abundant in the NO/GP-treated group. Conversely, granulation and large numbers of immune cells were observed in GP-treated and untreated groups. The amount of collagen in wound samples was visualized by Masson’s trichrome staining (blue color indicates collagen). As shown in Figure 8, samples from the NO/GP-treated group exhibited a prominent blue color similar to that of healthy skin tissue. However, GP-treated and untreated groups exhibited less collagen in the dermis region. Since inflammation was ongoing in these groups, the collagen synthesis and tissue remodeling processes were inhibited, resulting in delayed wound healing.

## 4. Conclusions

**Figure 9:**
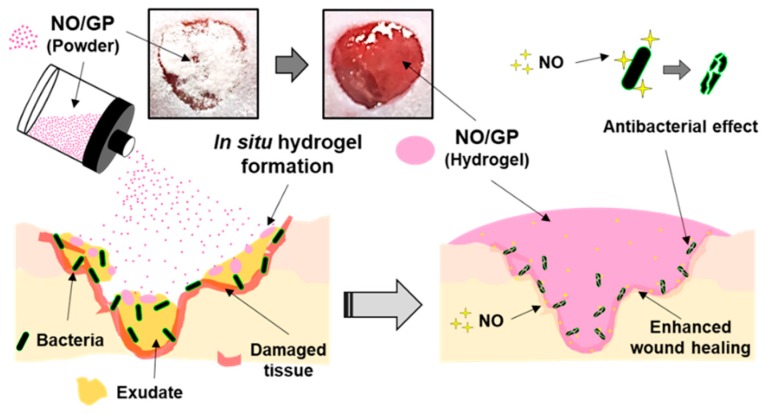
Schematic illustration of NO/GP for the treatment of infected wounds.

In this study, we successfully developed an in situ hydrogel-forming/NO-releasing wound dressing (NO/GP) composed of alginate, pectin, PEG, and GSNO, with a controlled NO release property and good storage stability for the effective treatment of infected wounds (Figure 9). Since NO/GP maintained a water-free powder form until use on the wound, the degradation of GSNO in NO/GP was prevented for more than 3 months when stored at 4 and 37 °C. When applied to wounds, NO/GP absorbed up to 350% of wound fluid and was quickly transformed from a dry powder to an adhesive hydrogel. Simultaneously, a NO release was triggered by absorbed wound exudates, followed by a sustained NO release over 24 h without an initial burst release. Rheological studies indicated that the hydrogel structure of NO/GP exhibited sufficient adhesiveness to remain stable on the wound surface. The results of an in vitro antibacterial study demonstrated that NO/GP leads to a 6-log reduction in MRSA and *P. aeruginosa* over 24 h. Finally, in vivo antibacterial effects and accelerated wound healing were observed in mice with infected wounds treated with NO/GP. These results suggest that the in situ hydrogel-forming/NO releasing formulation presented in this study can be fabricated by a simple and cost-effective manufacturing process and thus would be a promising alternative to dressings for the treatment of infected wounds.

## Figures and Tables

**Table 1:** Content homogeneity and flowability of in situ hydrogel-forming/nitric oxide (NO)-releasing powder dressing (NO/GP).

	Particle Size (μm)	GSNO Contents (%)	RSD of GSNO Contents (%)	Hausner Ratio
**Non-micronized mixture**	85.2 ± 47.3	4 ± 0.64	16.06	1.34 ± 0.04
**NO/GP**	51.5 ± 22	4 ± 0.19	4.77	1.97 ± 0.06

Particle size was analyzed by ImageJ software from microscopic images (*n* > 100). S-nitrosoglutathione (GSNO) contents and relative standard deviation (RSD) were calculated from 10 samples of NO/GP. The Hausner ratio was calculated from the tap/bulk density ratio (*n* = 3).

## References

[1] Daeschlein G. (2013). Antimicrobial and antiseptic strategies in wound management. Int. Wound J..

[2] Fleischmann C., Scherag A., Adhikari N.K., Hartog C.S., Tsaganos T., Schlattmann P., Angus D.C., Reinhart K. (2016). Assessment of global incidence and mortality of hospital-treated sepsis. Current estimates and limitations. Am. J. Respir. Crit. Care Med..

[3] Bello Y.M., Phillips T.J. (2000). Recent advances in wound healing. JAMA.

[4] Muzzarelli R.A. (2009). Chitins and chitosans for the repair of wounded skin, nerve, cartilage and bone. Carbohydr. Polym..

[5] Janis J., Attinger C. (2006). The basic science of wound healing. Plast. Reconstr. Surg..

[6] Witte M.B., Barbul A. (1997). General principles of wound healing. Surg. Clin. North Am..

[7] Yurt R.W., McManus A.T., Mason A.D., Pruitt B.A. (1984). Increased susceptibility to infection related to extent of burn injury. Arch. Surg..

[8] Percival S.L., Hill K.E., Williams D.W., Hooper S.J., Thomas D.W., Costerton J.W. (2012). A review of the scientific evidence for biofilms in wounds. Wound Repair Regen..

[9] Kevil C.G., Kolluru G.K., Pattillo C.B., Giordano T. (2011). Inorganic nitrite therapy: Historical perspective and future directions. Free Radic. Biol. Med..

[10] Schäffer M.R., Tantry U., Gross S.S., Wasserkrug H.L., Barbul A. (1996). Nitric oxide regulates wound healing. J. Surg. Res..

[11] Fatima A.D., Modolo L.V., Conegero Sanches A.C., Porto R.R. (2008). Wound healing agents: The role of natural and non-natural products in drug development. Mini Rev. Med. Chem..

[12] Rizk M., Witte M.B., Barbul A. (2004). Nitric oxide and wound healing. World J. Surg..

[13] Witte M.B., Barbul A. (2002). Role of nitric oxide in wound repair. Am. J. Surg..

[14] Jones M.L., Ganopolsky J.G., Labbé A., Wahl C., Prakash S. (2010). Antimicrobial properties of nitric oxide and its application in antimicrobial formulations and medical devices. Appl. Microbiol. Biotechnol..

[15] Schairer D.O., Chouake J.S., Nosanchuk J.D., Friedman A.J. (2012). The potential of nitric oxide releasing therapies as antimicrobial agents. Virulence.

[16] Privett B.J., Broadnax A.D., Bauman S.J., Riccio D.A., Schoenfisch M.H. (2012). Examination of bacterial resistance to exogenous nitric oxide. Nitric Oxide.

[17] Balakrishnan B., Mohanty M., Umashankar P., Jayakrishnan A. (2005). Evaluation of an in situ forming hydrogel wound dressing based on oxidized alginate and gelatin. Biomaterials.

[18] De Cicco F., Reverchon E., Adami R., Auriemma G., Russo P., Calabrese E.C., Porta A., Aquino R.P., Del Gaudio P. (2014). In situ forming antibacterial dextran blend hydrogel for wound dressing: SAA technology vs. spray drying. Carbohydr. Polym..

[19] De Cicco F., Porta A., Sansone F., Aquino R.P., Del Gaudio P. (2014). Nanospray technology for an in situ gelling nanoparticulate powder as a wound dressing. Int. J. Pharm..

[20] Dabiri G., Damstetter E., Phillips T. (2016). Choosing a wound dressing based on common wound characteristics. Adv. Wound Care.

[21] Del Gaudio P., Amante C., Civale R., Bizzarro V., Petrella A., Pepe G., Campiglia P., Russo P., Aquino R.P. (2019). In situ gelling alginate-pectin blend particles loaded with Ac2-26: A new weapon to improve wound care armamentarium. Carbohydr. Polym..

[22] Carpenter A.W., Schoenfisch M.H. (2012). Nitric oxide release: Part II. Therapeutic applications. Chem. Soc. Rev..

[23] Broniowska K.A., Diers A.R., Hogg N. (2013). S-nitrosoglutathione. Biochim. Biophys. Acta.

[24] Kannon G.A., Garrett A.B. (1995). Moist wound healing with occlusive dressings: A clinical review. Dermatol. Surg..

[25] Yoo J.W., Acharya G., Lee C.H. (2009). In vivo evaluation of vaginal films for mucosal delivery of nitric oxide. Biomaterials.

[26] Romic M.D., Klaric M.S., Lovric J., Pepic I., Cetina-Cizmek B., Filipovic-Grcic J., Hafner A. (2016). Melatonin-loaded chitosan/Pluronic(R) F127 microspheres as in situ forming hydrogel: An innovative antimicrobial wound dressing. Eur. J. Pharm. Biopharm..

[27] Balakrishnan P., Park E.K., Song C.K., Ko H.J., Hahn T.W., Song K.W., Cho H.J. (2015). Carbopol-incorporated thermoreversible gel for intranasal drug delivery. Molecules.

[28] Cho H.J., Balakrishnan P., Park E.K., Song K.W., Hong S.S., Jang T.Y., Kim K.S., Chung S.J., Shim C.K., Kim D.D. (2011). Poloxamer/cyclodextrin/chitosan-based thermoreversible gel for intranasal delivery of fexofenadine hydrochloride. J. Pharm. Sci..

[29] Kwak M.S., Ahn H.J., Song K.W. (2015). Rheological investigation of body cream and body lotion in actual application conditions. Korea-Aust. Rheol. J..

[30] Lin S.Y., Chen K.S., Run-Chu L. (2001). Design and evaluation of drug-loaded wound dressing having thermoresponsive, adhesive, absorptive and easy peeling properties. Biomaterials.

[31] Homsy C.A. (1970). Bio-Compatibility in selection of materials for implantation. J. Biomed. Mater. Res..

[32] Phaechamud T., Lertsuphotvanit N., Issarayungyuen P., Chantadee T. (2019). Design, fabrication and characterization of xanthan gum/liquid-loaded porous natural rubber film. J. Pharm. Investig..

[33] Levin R.J. (1992). The mechanisms of human female sexual arousal. Annu. Rev. Sex Res..

[34] Pearson J.P., Pesci E.C., Iglewski B.H. (1997). Roles of Pseudomonas aeruginosa las and rhl quorum-sensing systems in control of elastase and rhamnolipid biosynthesis genes. J. Bacteriol..

[35] McDougal L.K., Steward C.D., Killgore G.E., Chaitram J.M., McAllister S.K., Tenover F.C. (2003). Pulsed-field gel electrophoresis typing of oxacillin-resistant Staphylococcus aureus isolates from the United States: Establishing a national database. J. Clin. Microbiol..

[36] Castro J., Rivera D., Franco L.A. (2019). Topical anti-inflammatory activity in TPA-induced mouse ear edema model and in vitro antibacterial properties of Cordia alba flowers. J. Pharm. Investig..

[37] Rojas I.G., Padgett D.A., Sheridan J.F., Marucha P.T. (2002). Stress-induced susceptibility to bacterial infection during cutaneous wound healing. Brain Behav. Immun..

[38] Kominos S.D., Copeland C.E., Grosiak B., Postic B. (1972). Introduction of Pseudomonas aeruginosa into a hospital via vegetables. Appl. Microbiol..

[39] Lebrun P., Krier F., Mantanus J., Grohganz H., Yang M., Rozet E., Boulanger B., Evrard B., Rantanen J., Hubert P. (2012). Design space approach in the optimization of the spray-drying process. Eur. J. Pharm. Biopharm..

[40] Park E.K., Song K.W. (2010). Rheological evaluation of petroleum jelly as a base material in ointment and cream formulations: Steady shear flow behavior. Arch. Pharmacal Res..

[41] Amadeu T.P., Seabra A.B., De Oliveira M.G., Costa A.M. (2007). S-nitrosoglutathione-containing hydrogel accelerates rat cutaneous wound repair. J. Eur. Acad. Dermatol. Venereol..

[42] Schanuel F.S., Santos K.S.R., Monte-Alto-Costa A., de Oliveira M.G. (2015). Combined nitric oxide-releasing poly (vinyl alcohol) film/F127 hydrogel for accelerating wound healing. Colloids Surf. B Biointerfaces.

[43] Georgii J., Amadeu T., Seabra A., de Oliveira M., Monte-Alto-Costa A. (2011). Topical S-nitrosoglutathione-releasing hydrogel improves healing of rat ischaemic wounds. J. Tissue Eng. Regen. Med..

[44] Champeau M., Póvoa V., Militão L., Cabrini F.M., Picheth G.F., Meneau F., Jara C.P., de Araujo E.P., de Oliveira M.G. (2018). Supramolecular poly(acrylic acid)/F127 hydrogel with hydration-controlled nitric oxide release for enhancing wound healing. Acta Biomater..

[45] Herk L., Feld M., Szwarc M. (1961). Studies of “cage” reactions. J. Am. Chem. Soc..

[46] Shishido S.M., Oliveira M.G. (2000). Polyethylene glycol matrix reduces the rates of photochemical and thermal release of nitric oxide from S-nitroso-N-acetylcysteine. Photochem. Photobiol..

[47] De Oliveira M.G., Shishido S.M., Seabra A.B., Morgon N.H. (2002). Thermal stability of primary S-nitrosothiols: Roles of autocatalysis and structural effects on the rate of nitric oxide release. J. Phys. Chem. A.

[48] Hetrick E.M., Shin J.H., Paul H.S., Schoenfisch M.H. (2009). Anti-biofilm efficacy of nitric oxide-releasing silica nanoparticles. Biomaterials.

[49] Raulli R., McElhaney-Feser G., Hrabie J., Cihlar R. (2002). Antimicrobial properties of nitric oxide using diazeniumdiolates as the nitric oxide donor. Rec. Res. Dev. Microbiol..

[50] CHEN A.F. (2005). Nitric oxide: A newly discovered function on wound healing. Acta Pharmacol. Sin..

[51] Heggers J.P., Robson M.C., Doran E.T. (1969). Quantitative assessment of bacterial contamination of open wounds by a slide technique. Trans. R. Soc. Trop. Med. Hyg..

[52] Robson M.C. (1997). Wound infection: A failure of wound healing caused by an imbalance of bacteria. Surg. Clin. N. Am..

